# Analysis of the Acoustic Transcranial Bone Conduction

**DOI:** 10.3390/audiolres12020019

**Published:** 2022-03-26

**Authors:** Catherine Dufour-Fournier, Arnaud Devèze, Jonathan Barbut, Erick Ogam, Issam Saliba, Catherine Masson

**Affiliations:** 1 Department of Otolaryngology and Head and Neck Surgery, University of Montreal, Montreal, QC H3T 1J4, Canada; c.dufour.fournier@gmail.com; 2 Department of Otology and Skull Base Surgery, Ramsay Health Care, Clairval Hospital, 317 Bd du Redon, 13009 Marseille, France; dr.deveze@gmail.com; 3 Laboratory of Applied Biomechanisms, Faculty of Medicine, Aix-Marseille University, Bd Pierre Dramard, 13015 Marseille, France; jonathan.barbut@gmail.com (J.B.); catherine.masson@univ-eiffel.fr (C.M.); 4 Laboratory of Applied Biomechanisms, Gustave Eiffel University, Bd Pierre Dramard, 13015 Marseille, France; 5 Department of Otolaryngology and Head and Neck Surgery, Sainte Musse Hospital, Rue Henri Sainte Claire Deville, 83100 Toulon, France; 6 Laboratory of Mechanics and Acoustics, Aix Marseille University, CNRS, Centrale Marseille, LMA UMR 7031, 13015 Marseille, France; ogam.erick@gmail.com; 7 Department of Otolaryngology and Head and Neck Surgery, University of Montreal—CHUM, 1051 Sanguinet Street, Montreal, QC H2X 3E4, Canada

**Keywords:** otology, sound transmission, acoustic, bone conduction, BAHA

## Abstract

Objectives: (1) To analyze the preferential pathways of sound transmission and sound waves travelling properties in the skull and (2) to identify the location(s) on the skull where bone conduction to the cochlea is optimal. Study design: Basic research Methods: Nine cadaveric heads were placed in an anechoic chamber and equipped with six Bone Anchored Hearing Aids (BAHA™) implants (Cochlear™, Sydney, NSW, Australia) and fifteen accelerometers. A laser velocimeter was used to measure cochlear response by placing a reflector on the round window. Different frequency sweeps were applied to each implant, and measurements were recorded simultaneously by the laser velocimeter and accelerometers. Results: Low-frequency sound waves mostly travel the frontal transmission pathways, and there is no clear predominant pattern for the high frequencies. The mean inter-aural time lag is 0.1 ms. Optimal sound transmission to the cochlea occurs between 1000 and 2500 Hz with a contralateral 5 to 10 dB attenuation. The implant location does not influence mean transmission to the cochlea. Conclusion: There is a pattern of transmission for low frequencies through a frontal pathway but none for high frequencies. We were also able to demonstrate that the localization of the BAHA™ implant on the skull had no significant impact on the sound transmission, either ipsi or contralaterally.

## 1. Introduction

Conductive hearing loss is found in diseased or malformed external or middle ears and can cause a hearing loss up to 60 dB HL [1]. Conditions like otosclerosis or anatomical anomalies can sometimes be addressed surgically, but technical difficulties or refusal of the patient to undergo an operation can be obstacles to treatment. Historically, tuning forks were used to help in the diagnosis of hearing loss, using the comparison of air and bone sound transmission [2]. More recently, bone conduction devices and implants have been used to improve the hearing of people suffering from conductive hearing loss, whatever the initial cause of the condition. Clinical experience and research have proved the efficacy of this technology [3]. However, not many fundamental studies were conducted on the pathways of sound conduction in the skull, a topic relevant to the future optimization of the use of bone implants. Studies have theorized about how the sound itself was transmitted to the basilar membrane of the inner ear through the skull, explaining the basic functioning of bone conduction [4,5]. Different stimulation points on the skull with the corresponding response of accelerometers and the cochlea were also studied, but the locations tested (temporal, parietal, and frontal bone) were not compatible with finding new opportunities for implant localization improvement [6,7].

This study had two different aims. The first was to identify the location or locations on the skull where bone conduction to the cochlea is optimal by using three different arrangements of implants on each side that could be used in actual practice. The second goal was to determine the frequency, trajectory, and timing properties of the preferential pathways of a sound wave through the skull.

## 2. Materials and Methods

The experiments were conducted at the Laboratory of Applied Biomechanisms in Aix Marseille University, France. Nine cadaveric heads were acquired and used in the course of this experiment, preserved in Winckler’s solution in a cold room. They were de-identified, and no demographic data were available about the cadavers. Approval for the study was not required in accordance with local and national legislation.

**Figure 1:**
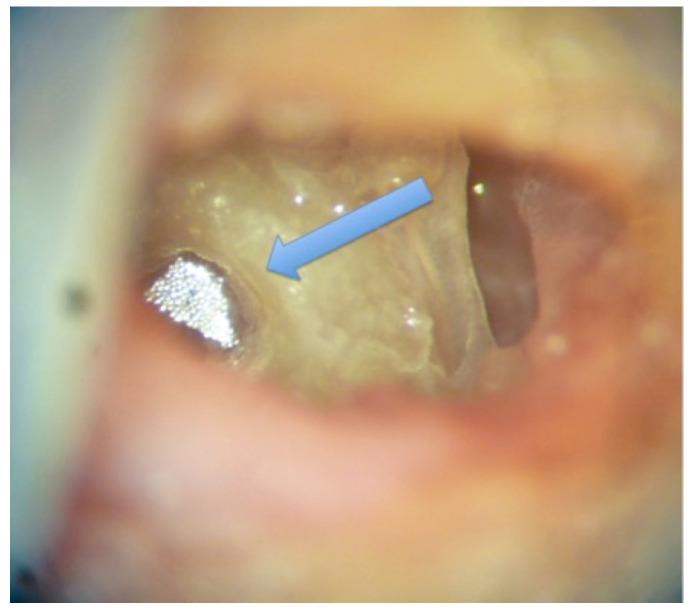
Reflector on the round window (white color). The blue arrow shows the round window.

A standard bilateral mastoidectomy, including a facial recess approach to access the round window, was performed on each head. The edge of the round window niche was slightly drilled to have direct access to the round window membrane. A 1 mm^2^ reflector for the laser beam of the velocimeter was placed on the round window on one side (Figure 1).

**Figure 2:**
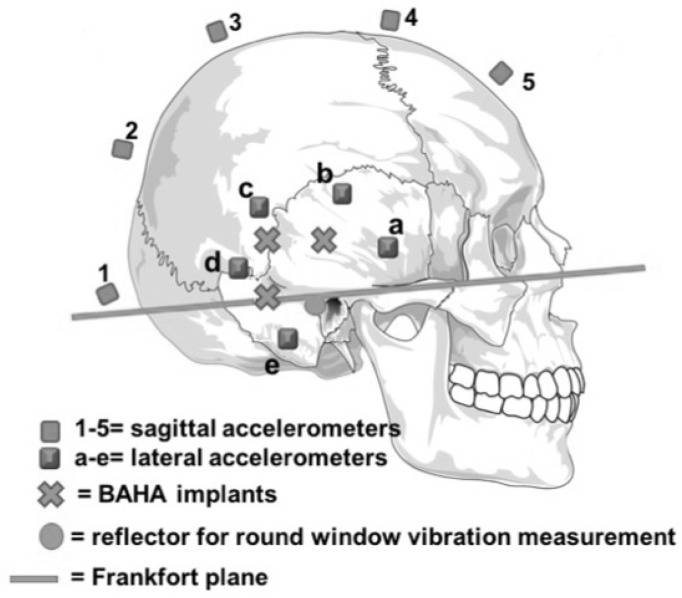
Positions of the three BAHA^TM^ implants (crosses), the five accelerometers set along the sagittal line (1–5), the five accelerometers set around each ear (a–e), the reflector deposited on the round window for use with the laser velocimeter (disk) and the Frankfort plane (straight line).

The pinna and the soft tissue behind it were removed on each side to adequately expose the mastoid bone and the space needed for the implants. BAHA™ implants (Cochlear™, Sydney, NSW, Australia) were placed at three different locations bilaterally. They were all set at the same distance (2.5 cm) from the external auditory canal at 0°, 45°, and 90° from the Frankfort plane (Figure 2), a virtual line passing through the external auditory canal and extending to the inferior limit of the orbital rim.

**Figure 3:**
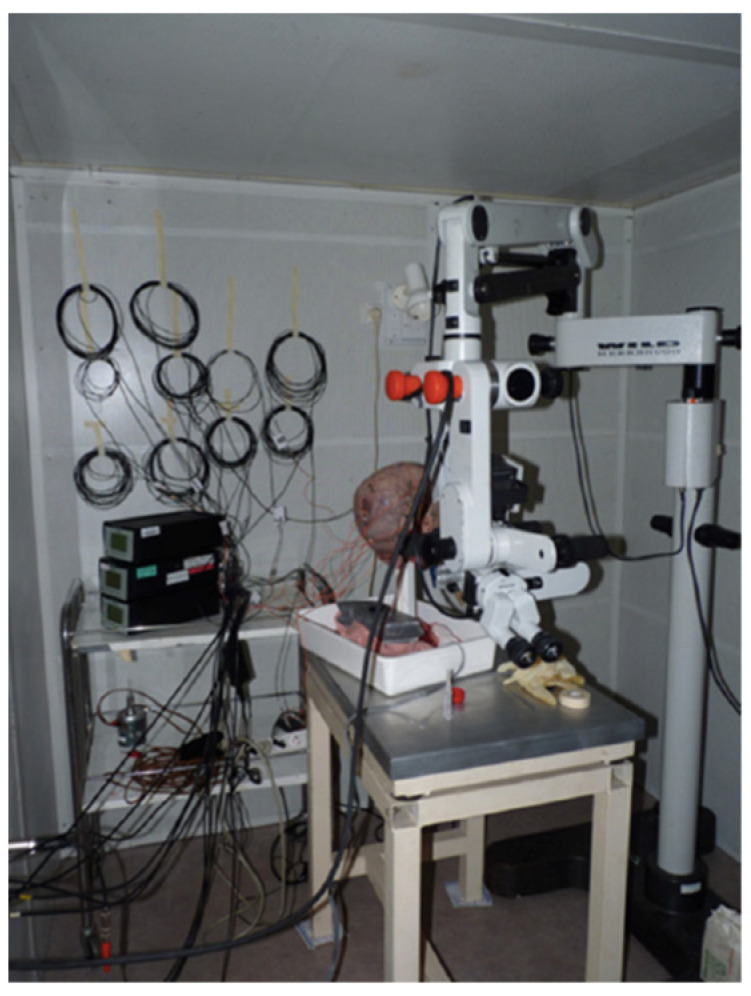
Experiment set-up in the anechoic chamber.

The implants were fixed in the bone with a measured strength of 45 Newtons. Fifteen linear accelerometers (Bruel and Kjaer 45–17c, Naerum, Denmark) were installed: Five accelerometers were placed at equal distance on the sagittal midline from frontal to occipital, one beside each BAHA™ implant (three on each side) and one in each of the spaces available between the implants (two on each side). To fix all the accelerometers, we exposed the bone by dissecting five squares of soft tissue (2 × 2 cm). The accelerometers were glued directly on the bone for optimal data monitoring. Linear accelerometers, which measure acceleration in only one direction, were used to measure the linear vibration passing through them in the coronal plane from the emitting ear to the other ear. To avoid any damping of the vibrations by external contact and to maintain the head in an upright fixed known position for the duration of the experiment, a stake was placed through the medullar canal of the remaining cervical vertebrae. The microscope, head, and laser were all placed on anti-vibration tables in an anechoic chamber (Figure 3).

The laser velocimeter (Laser Doppler Vibrometer OFV 534-Polytec, Hudson, MA, USA) was oriented so as to send a signal directly on the reflector on the round window on one side. The laser, accelerometers, and BAHA™ implants interfaced with an automatic Ultrasonic Pulse Velocity (UPV) 8-channels audio analyzer (Rohde-Schwarz, Attleboro, MA, USA). Each BAHA™ implant was tested in sequence. The UPV transmitter covered a frequency range from 100 Hz to 10 kHz per sweep, which are frequencies relevant to human hearing [8]. The response of each accelerometer and of the laser velocimeter was measured and recorded separately. The UPV transmitter was used to transmit the aforementioned frequency range sweep three times to each implant. Two complete frequency sweep response measurements, in addition to one timing measurement (inter-aural time difference), were recorded. The linear accelerometers’ recordings were done randomly with emission from only one side, followed by the contralateral side. The round window response was measured for both the ipsi and contralateral emissions to get data on bilateral stimulations.

The data from the UPV were normalized over frequency bins corresponding to ^1^/_3_ of an octave per data point by MATLAB scripts (The MatWorks, Natick, MA, USA). An Analysis of Variance (ANOVA) was performed on the data sets collected at 500 and 8000 Hz. The Tukey HSD (honest significant difference) [9] test that uses the results of the ANOVA to distinguish if the means are different from one another to a significance level *p* < 0.05 was applied to all the data sets.

## 3. Results

### 3.1. Propagation Properties

**Figure 4:**
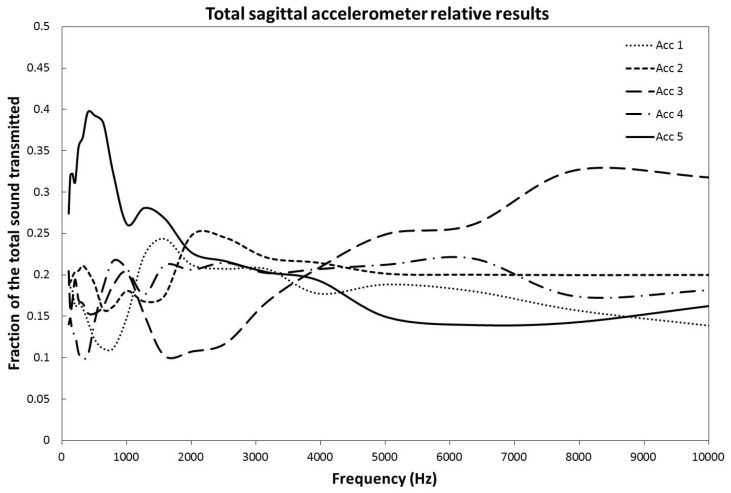
Relative fraction of the signal transmitted across the sagittal line by each of the five accelerometers set along it as a function of the implant signal frequency. The results have been averaged over all orientations of the BAHA^TM^ implants about the Frankfort plane.

The propagation properties were analyzed by comparing the results of the different sagittal linear accelerometers. To obtain a reproducible measure from head to head and to account for morphological differences, the recorded signal of each sagittal accelerometer was divided by the sum of the signals from all the sagittal accelerometers (Figure 4). This gives the fraction (%) of the initial sound signal stimulating each accelerometer. The signal was sent as a continuous sweep of frequencies, however, the data are presented as the mean value over a scale of ^1^/_3_ octave against the mean frequency of each ^1^/_3_ octave. We used the ^1^/_3_ octave scale because it corresponds to the scale used in acoustic engineering as a match for human perception [10].

Independent of the location of the implant, the low frequencies (100 to 1500 Hz) are preferentially transmitted via the most frontally located accelerometer (#5). With a zero degree placed BAHA™ implant, the frontal accelerometer recorded a peak of 36% of all the transmission at 1272 Hz, 47% of all transmission at 396.5 Hz for a 45 degrees’ placement and 42% at 502 Hz for a 90 degrees’ placement. The average overall peak was 40% of the signal at 396.5 Hz. In the mid-range frequencies, from 1500 to 5000 Hz, no dominance of transmission can be clearly established. In the uppermost frequencies analyzed, a dominance of transmission is observed from accelerator #3 situated on the superior part of the skull.

The ANOVA and Tukey HSD tests were performed on the 500 and 8000 Hz data. These frequency areas have, as a preferred pathway, the zone around accelerometer #5 for 500 Hz and accelerometer #3 for 8000 Hz. For the 0, 45, and 90 degrees positioning of the implant, as well as for the combined set of data at 500 Hz, the differences of transmission between accelerometer 5 and the average of the other four accelerometers were of 18% (*p* = 0.047), 27% (*p* < 0.001), 28% (*p* = 0.029), and 24% (*p* < 0.001), respectively. These results were all considered statistically significant. For the 8000 Hz range, the differences of transmission between accelerometer #3 and the average of the other four accelerometers were of 13% (*p* = 0.34), 9% (*p* = 0.37), 25% (*p* < 0.001), and 16% (*p* < 0.001) for the 0, 45, 90 degrees and combined set, respectively. The *p* value was only statistically significant for the 90 degrees and combined set data [11] (Table 1).

### 3.2. Analysis of the Timing Response

**Figure 5:**
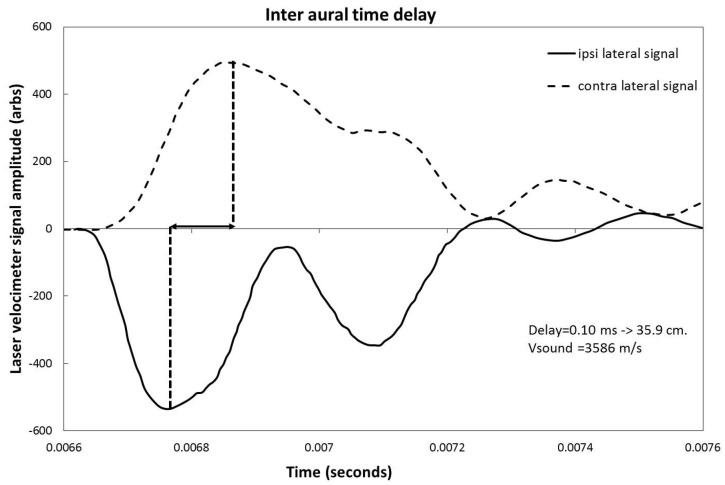
Measurement by laser velocimeter of the inter-aural time response of the round window driven by a 220 μs square pulse. For better visualization, the ipsilateral signal (full line) has been inverted with respect to the contralateral signal (dashed line).

The time response of the cochlea to a sharp 221 µs impulse was measured with the laser velocimeter for the ipsilateral and contralateral cases. Figure 5 shows the signals and their time delays in the case of the 0° implant. For clarity, the polarity of the signal from the ipsilateral cochlea is inverted. The measured peak-to-peak time delay is 0.1 ms. Assuming a mean sound speed of 3586 m/s in cortical bone [12], this corresponds to a distance of 35.9 cm. The signals from the contralateral ear also show clear evidence of damping and pulse broadening due to transmission effects across the skull.

### 3.3. Cochlear Transmission

To calculate the cochlear transmission, the laser velocimeter was used to directly measure the response of the round window to low (100 to 4000 Hz) and high (4000 to 10,000 Hz) frequency sweeps of 220 ms duration. The displacement speed of the window was the value measured. The amplitude of the signal indicates the magnitude of the movement of the cochlear fluids in the internal ear for each frequency.

**Figure 6:**
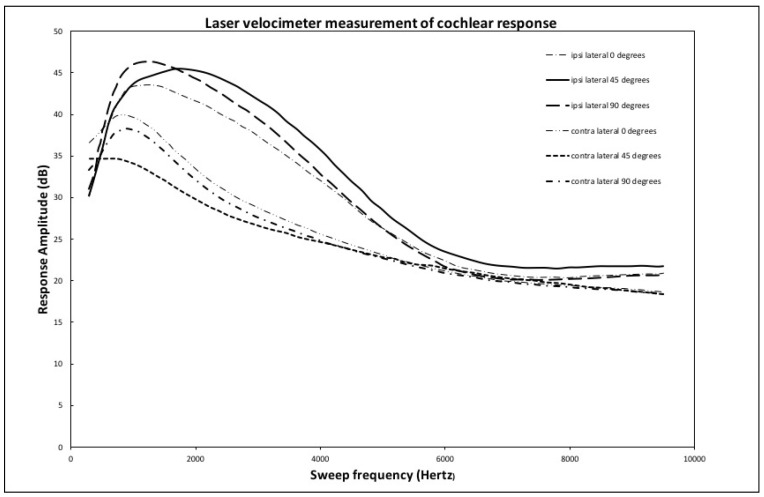
Ipsilateral and contralateral averaged round window response: 0 to 10,000 Hz.

The graph in Figure 6 shows the average for all heads of the frequency response in both ipsilateral and contralateral cases for each of the implant locations. The initial noisy data for each curve was smoothed by performing a moving frequency average over a band of 300 Hz. This smoothed data set was subsequently averaged over all tested heads. For ease of visualization, a normalization factor of 50 was used to multiply the data. This is equivalent to an upward shift of 33 dB in the zero-reference signal level. All the statistical analyses were carried out on the original data, and this normalization obviously does not affect any conclusions as to the relative amplification levels of the signals with respect to one another.

The ipsilateral cases show, for each complete frequency sweep, a response peaking in a zone extending from 1000 to 2500 Hz with subsequent slow damping of the higher frequencies. For the contralateral cases, the peak response is lower by 5 to 10 dB and happens at lower frequencies between 500 and 1200 Hz. This behavior of the average values of the cochlear frequency responses is consistent with the results of the analysis of the timing signals. It is important to note that, for the average over all heads, no significant difference exists in the transmission of the signal to the cochlear fluids coming from the different locations of the sound source, whether at 0, 45, or 90 degrees from the Frankfort plane.

## 4. Discussion

In this study, we have been able to show that the frontal pathway significantly dominates transmission for all implants separately and for the combined locations at lower frequencies. This means that the implant location has no significant effect on side-to-side transmission at those frequencies in a statistically significant manner. However, at higher frequencies, the results on propagation properties were not as clear-cut. Even though we can clearly appreciate, in Figure 4, that above 7000 Hz there seems to be a clear predominance of accelerator #3, only the 90 degrees and combined signals were transmitting preferentially through the superior part of the skull with a statistically significant *p* value of less than 0.05 (*p* < 0.001). Taken alone, the 0 and 45 degrees BAHA™ implants seemed to show a tendency to use that pathway, although not in a statistically significant manner. Figure 4 shows the results for the combination of all implant placements. It is statistically significant due to the very low *p* value of the 90 degrees set (*p* < 0.001), which influences the mean in a disproportionate way. There is no clear explanation of why the signal transmits that way. We hypothesize that lower frequencies signals adopt a more frontal pathway because the frontal sinus might be creating a less dense region with more resonance, advantaging the transfer of those frequencies. We could argue that the presence of the mastoid posteriorly could play a similar role [13], but the frontal sinus is more aerated than the mastoid, and all three implants’ locations are on or above the Frankfort plane, therefore above the plane of most of the mastoid region. For the higher frequencies, accelerator #3 is the closest to the vertex, and there is no aeration in the upper skull, optimizing transmission at higher frequencies.

As mentioned in the results section, the laser velocimeter frequency sweep shows a response that peaks in the 1000 to 2500 Hz zone for the ipsilateral case, while this same peak is damped by 5 to 10 dB and shifted to lower frequencies for the contralateral side. This effect is due to the transmission response of the skull that both shifts frequency and damps the signal from the implant. The same effect is also visible in the time response curves shown in Figure 5. Even though some small differences in average response can be seen between the implant location for both the ipsi and contralateral cases, no statistically significant differences that would favor a particular implant location are shown.

Other studies [6,7] tested implant locations not applicable in a clinical setting. This experiment tested potential locations that could be used on patients for surgical implantation. Thus, the data collected could be relevant in the greater aim of improving vibratory implant auditory results.

It is important to note the potential effects of using cadaveric heads. No osseointegration of the implant of the BAHA™ is possible, leaving open the possibility of a slightly weaker and less than optimal sound transmission to the skull from the vibration of the implant. However, the implant is already in firm mechanical contact with the skull by itself, as we could detect from the large signal amplitudes observed in the experiment. The end results should, therefore, be unaffected by this limitation. The second limitation comes from dehydration of the tissues, especially the cochlear fluids. It is very difficult to know or extrapolate the effect of brain dehydration on the transmission in the skull and the cochlea [14]. In living persons, the brain and its fluid, which are in contact with the whole cranial vault, most probably drive a part of the bone-conducted cues. However, in cadavers, the dehydration of the brain may result in an air interface underneath the skull that may impact or dampen the sound wave transmission. For the cochlea, the limitation is expected to be a weaker transmission to the velocimeter due to less fluid being available to make the round window move when stimulated with sound. If no liquid was present in the cochlea, the velocimeter would have picked-up air displacement, which is similar to liquid displacement although weaker. As with the non-ossification of the BAHA™, this attenuation would not change the relative results but only slightly diminish the signal amplitude. The skin needed to be removed to place the implants and accelerometers. We minimized the skin removed to make sure the pathways taken by the vibrations were mostly intact. Finally, the embalmment and preservation of the head itself is a limitation. Mc Elhaney et al. studied the effect of embalmment on bones and noted generally for all methods a loss of compressive strength and an increase in hardness [15]. Since the change in property would apply to all the bones, it could affect the raw results numbers but not the interpretation of those numbers.

We compared our results with data observed in a clinical and experimental context [16]. As noted previously, the interaural attenuation was of 5 to 10 dB, as would be expected in clinic. The time delay response was also compatible when converted into a distance in cm, with the known speed of transmission of sound in bone [12]. Those parameters confirmed that the data collected are relevant and will be comparable to true clinical features.

While the averaged data presented in the results section show strong regularities, there is still a notable morphology-dependent effect on the different heads. This implies that further studies on the different types of morphologies and what they imply in terms of optimal implant location need to be pursued, using the interaural time signals and the ratio of contra to ipsilateral frequency sweeps to obtain individual skull response functions is an example. In the future, a good understanding of the pathways of transmission and the significant morphological features related to them could lead to the possibility of a tailored selection of device and implantation location for each patient. During the experiment, we also scanned and modelized the heads. The present data set, as it includes both time and frequency response spectra and a CT-scan of each head, could be used in the future to build and verify a finite-element model of the skull in order to propose a virtual benchmark for the development of further bone conductions devices.

Throughout this study, great care was taken to ensure the reproducibility of the results. The work was carried out in the best possible audio conditions to minimize artifact signals. Using an anechoic chamber, anti-vibration tables, and a spine fixation considerably reduced the likelihood of spurious audio signals corrupting the data. This technique ensured reproducibility of the results by guaranteeing consistent and minimal damping of vibrations.

The data were presented and analyzed in a way that would allow other researchers to easily compare their results to ours. An example is the use of fractions in the analysis of the sound transmission pathways through the coronal plane of the skull. With this technique, any type of sound transmitter and vibration receptor could be used as long as they are set in the pattern described in the methods section. The results, when normalized as a fraction of the total sound transmission through the sagittal accelerometers, could be directly compared to the present work.

## 5. Conclusions

We were able to demonstrate that the main pathway of sound transmission was through the frontal pathway for lower frequencies and that there was no clear pathway at high frequencies. We also specified the relative importance of all the other pathways in relation to one another. Our measured transmission properties were consistent with the clinical data. Our experiment showed that for all three locations tested with the implants, none was significantly better at transmitting sound to the cochlea either ipsilaterally or contralaterally.

## Figures and Tables

**Table 1:** Propagation properties of accelerators 5 and 3.

Accelerator 5–500 Hz			Accelerator 3–8000 Hz		
BAHA Implant Position	Transmission Difference (%)	*p* Value	BAHA Implant	Transmission Difference (%)	*p* Value
0°	18	0.047	0°	13	0.34
45°	27	<0.001	45°	9	0.37
90°	28	0.029	90°	25	<0.001
Combined	24	<0.001	Combined	16	<0.001

## Data Availability

Not applicable.
